# Three Destinies of Solution-Processable Polymer Light-Emitting Diodes under Long-Time Operation

**DOI:** 10.3390/polym13111853

**Published:** 2021-06-02

**Authors:** Ruslana Udovytska, Pavel Chulkin, Aleksandra Wypych-Puszkarz, Jaroslaw Jung

**Affiliations:** 1 Department of Molecular Physics, Faculty of Chemistry, Lodz University of Technology, Zeromskiego 116, 90-924 Lodz, Poland; ruslana.udovytska@p.lodz.pl (R.U.); aleksandra.wypych@p.lodz.pl (A.W.-P.); 2 Faculty of Chemistry, Silesian University of Technology, Strzody 9 str., 44-100 Gliwice, Poland; pavel.chulkin@polsl.pl

**Keywords:** polymer light-emitting diodes, super yellow, organic electronics, impedance spectroscopy, equivalent circuit

## Abstract

The article describes three different ways of polymer light-emitting diode (PLED) degradation, caused by damage of the protective layer. The electroluminescence and charge-transport properties of a completely encapsulated diode, the diodes with a leaky protective layer and diodes without encapsulation were compared under long-time exploitation. The studied devices incorporated Super Yellow light-emitting poly-(1,4-phenylenevinylene) PPV copolymer as an electroluminescence component, and (poly-(3,4-ethylenedioxythiophene)–poly-(styrene sulfonate) (PEDOT:PSS) as a charge-transport layer between the indium tin oxide (ITO) anode and aluminum–calcium cathode. To analyze the PLED degradation mechanism regarding charge transport, impedance spectroscopy was used. The values of resistance and capacitance of the internal layers revealed an effect of applied voltage on charge carrier injection and recombination. The factors responsible for the device degradation were analyzed on a macromolecular level by comparing the plots of voltage dependence of resistance and capacitance at different operation times elapsed.

## 1. Introduction

Research and development in the field of PLEDs are progressing rapidly, broadening their application in head-up car dashboards, billboards, home and office lighting, and flexible displays. Compared to their inorganic counterparts, PLEDs with thin organic layers are lighter, more flexible and use less energy. However, the wide application of PLEDs is limited so far by several problems, such as lifetime and vulnerability to mechanical damage and chemical corrosion caused by water and oxygen penetration [1,2].

The PLED comprises of a stack of organic layers sandwiched between an anode and a cathode. One of the basic PLED configurations contains indium tin oxide (ITO) [3] used as a transparent anode. The second layer is made of PEDOT:PSS (composite of (poly-(3,4-ethylenedioxythiophene)–poly-(styrene sulfonate)) [4], which shows high conductivity and good transparency. It is the most widely used hole-transporting layer (HTL) in the organic electronic device structure. The relatively high value of work function of the PEDOT:PSS, equal to about 4.9 eV, is responsible for reducing the energetic barrier between the anode (ITO, work function about 5 eV) and the energy of the highest occupied molecular orbital (HOMO) of the next light-emitting layer (EL) [5,6]. The role of the EL is to convert the energy of the electric charge carriers to light via bimolecular recombination between the holes and electrons. Poly-(para-phenylene vinylene) copolymer, known as Super Yellow (SY) [7], is one of the most widely used conjugated polymers for solution-processable PLEDs [8]. An important and desirable feature of the Super Yellow is its high quantum efficiency, and similar charge mobility for both the holes and electrons [9]. Such a balanced ambipolar transport is crucial for an efficient PLED, although in most conjugated polymers the hole mobility is usually higher than the electron mobility. An electron transport layer (ETL) is deposited onto the SY. The good effect is obtained by using calcium (Ca) with the work function comparable to the LUMO level of the emitters made of SY. The last layer is a cathode made of aluminum (Al), which protects the very reactive Ca layer from the effects of the weather conditions. However, this method of device protection is not sufficient for PLEDs, so the aluminum is usually covered with the transparent plastic casing.

The degradation of PLEDs is the most serious obstacle towards their commercialization. The main reason for PLED ageing is the degradation that occurs when the device is exposed to moisture or oxygen [10]. The PLED degrades due to different mechanisms mainly caused by the instability of the low-work-function cathode, and pin-hole formation during fabrication, which provides a path for oxygen and moisture infiltration.

The presented work is devoted to the study of the transport of charge carriers in PLEDs with active SY layers. The analysis was carried out for the manufactured devices operating in full-encapsulation conditions and with a partially destroyed protective casing, as well as for the devices without a protective layer. The mechanisms responsible for the deterioration of the efficiency of polymer diodes, caused by the destructive action of water and oxygen penetrating the semiconductor/PEDOT: PSS/ITO interfaces, have been proposed for the first time.

## 2. Materials and Methods

**Figure 1:**
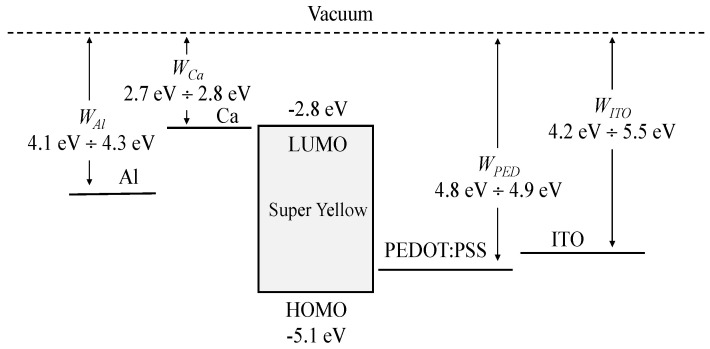
The energy of HOMO and LUMO levels of the emitting Super Yellow layer and work functions *W_Al_*, *W_Ca_*, *W_PED_* and *W_ITOl_* of calcium, aluminum PEDOT:PSS and ITO materials, respectively.

The PLED samples were fabricated in a glovebox system under dry nitrogen atmosphere at room temperature. The organic layers were deposited on glass slides pre-coated with ITO from Ossila (Sheffield, UK) with sheet resistance lower than 10 Ω/sq. The substrates were cleaned sequentially in an ultrasonic bath by isopropanol and acetone and then treated by oxygen plasma to remove organic residues. The polymer PEDOT:PSS Baytron P from H.C. Starck GmbH, Electronics and Optics Business Group (Leverkusen, Germany) was dissolved in methanol (1 mg per 1 mL) and was spin-coated onto the cleaned substrate. Subsequently, the emissive layer was deposited onto ITO/PEDOT:PSS substrate by spin coating of the SY solution in Toluene HPLC (1 mg per 5 mL). The organic layer thickness was 70 nm. A thin layer of Ca 15-nm thick was evaporated onto the SY surface and finally, the Ca sheet was coated with a 100-nm thick Al layer. The final structure of the fabricated PLEDs consisted of the following 5 layers: ITO/PEDOT:PSS/SY/Ca/Al. An energetic diagram of the devices is given in Figure 1.

The samples were encapsulated to protect the organic layers against oxygen and moisture. The LED encapsulation epoxy (E132) from Ossila (Sheffield, UK) as an adhesive and non-damaging material was used. Curing of epoxy resin was achieved at UV illumination at a wavelength of about 350 nm for 2 min. The resin in conjunction with a glass coverslip provided a robust barrier, ensuring an extended service life during measurement and storage.

Electrochemical measurements were carried out using Alpha Analyzer Novocontrol Technologies [11]. Impedance spectra were obtained in the 1.7 Hz–20 MHz frequency range (the number of frequencies in one spectrum was 37). The DC voltage was applied in a range from 0 V to 4 V. The working range of DC was extended in special cases described in the article. AC voltage overlapped the DC signal with an amplitude of 10 mV. Data files were analyzed and simulated with equivalent electrical circuits using EIS analyzer software [12]. The electroluminescence characteristics were measured using Ocean Optics spectrometer (365–900 nm).

## 3. Results and Discussion

The tested devices were exposed to a set of controlled destructive factors caused by environmental and exploitation conditions. The subsections below show the evolution of the optical and electrical response, which can be used as a measure of degradation.

### 3.1. The Fully Protected PLEDs

**Figure 2:**
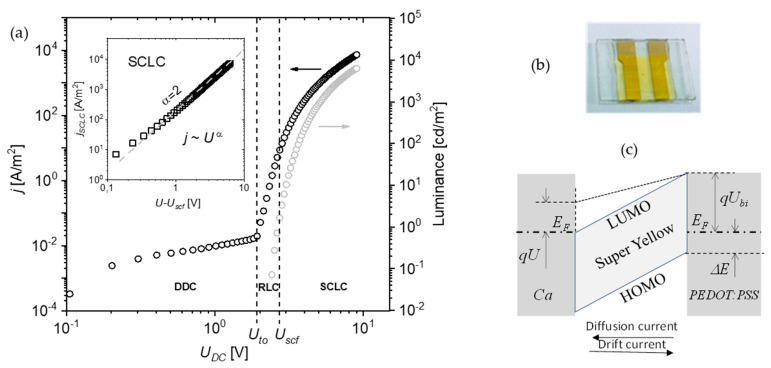
Current-voltage characteristic of the encapsulated PLED(ITO/PEDOT:PSS/SY/Ca/Al) (black circles), the luminance as a function of applied DC voltage (grey circles) and in the inset the space charge limited current (black squares; the vertical dashed lines indicate the boundary voltage values turn-on voltage *Uto* and space charge formation voltage *Uscf* limits: drift-diffusion current DDC, recombination-limited current RLC and space-limited current SCLC (**a**). Photo of the encapsulated PLED (**b**). An PLEDenergy diagram after electrode short-circuited and Fermi *E_F_* levels equalized; the symbols *q*, *Ubi* and Δ*E* denote the elementary charge, the built-in voltage and the energy barrier for injecting holes from the PEDOT electrode, respectively; thermal equilibrium and upon applying to calcium electrode the bias voltage (*UDC* < *Ubi*) is marked with dotted line (**c**).

**Figure 3:**
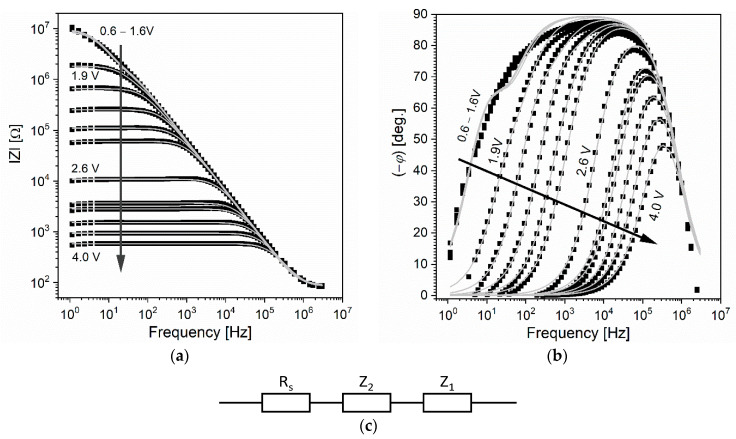
Set of impedance spectra of the PLED in the applied DC voltage range from 0.6 V to 4.0 V (black points) and example of fitting the spectra (grey line) to equivalent circuit model. Impedance module (**a**) and phase shift (**b**) as functions of frequency (Bode plots). Equivalent electric circuit of operating PLED including resistor *R_s_* = *R_S1_* + *R_S2_* and two elements with impedances *Z*_1_ and *Z*_2_ (**c**).

The turn-on voltage (*U_to_*) of the studied type of device was equal to about 1.9 V—the double-logarithmic graph reveals a noticeable break at this value, proving a significant increase in the device conductivity and switch-on of the emission. The turn-on voltage corresponds to the built-in voltage (*U_bi_*) resulting from the thermodynamic equilibrium of the system reached after the electrode is short-circuited. After equalizing the Fermi levels, the built-in voltage is formed and it is equal to the difference between the work function of the PEDOT:PSS (*W_PED_*) and calcium (*W_CA_*) electrodes divided by the elemental charge (q) (Figure 1 and Figure 2c). In other words, as soon as the turn-on voltage (*U_to_* ≈ *U_bi_*) is achieved, the mechanism of charge transport changes to facilitate the effective delivery and recombination of the charge carriers.To get a deeper insight into the nature of the process, the operating PLED was studied by impedance spectroscopy. As the important role of applied voltage has been noticed, the impedance spectra were registered in a wide voltage range, covering the turn-on region. The Nyquist type of plots, despite being the most popular to represent impedance data, appear not to be useful when one deals with voltage effect, which causes the impedance value to differ in several orders of magnitude. The Bode plots prove to be more useful to perceive the changes caused by voltage increase, providing the impedance module logarithm (|*Z*|) and the phase shift (*φ*) (1) as functions of AC frequency.

(1) $$\begin{array}{c} \left|Z\right|\;=\;\sqrt{Z_{re}^{2}+Z_{im}^{2}}\\ \varphi =\arctan\left(\frac{Z_{im}}{Z_{re}}\right) \end{array}$$

where *Z_re_* and *Z_im_* are real and imaginary parts of the impedance (Figure 3a,b).

An analysis of the graphs presented in Figure 3 allowed us to make the following conclusions: (i) Within the voltage range *U_DC_* less than 1.9 V, the system demonstrates a large impedance module at low frequencies (Figure 3a), and in the frequency range between 100 Hz and 30 kHz it has a capacitive behavior, which is indicated by the phase shift close to −90° (Figure 3b). (ii) Within the voltage range between 1.9 V and 2.6 V, the impedance module decreases about three orders of magnitude. The lowest frequency at which the device demonstrates capacitor-like behavior (phase shift equal to −90°) gradually increases, which means that the device loses its capacitive properties. (iii) At voltages higher than 2.6 V, the impedance still decreases with the voltage, yet not so drastically as within the previous range. The capacitive effects become considerably small (at *U_DC_* = 4 V, the phase shift does not exceed −50°). Based on those observations, one can assume that there are different mechanisms of charge transport that are switched by the voltage applied. In the first case, at *U_DC_* < 1.9 V, the charge carriers move slowly and localize in the SY layer, forming a non-compensated space charge. When 1.9 V < *U_DC_* < 2.6 V, the mobility of the charge carriers grows and the time required for the charge rearrangement becomes smaller. At *U_DC_* > 2.6 V the conductance growth is not so strong, whereas the charge carriers rearrange faster.

**Figure 4:**
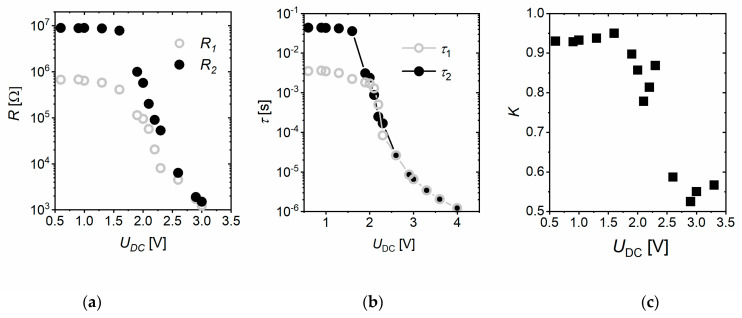
Calculated equivalent circuit resistance (**a**), time constants (**b**), and ratio between resistance as functions of applied voltage (**c**). According to the explanation given in the text, the indexes 1 and 2 correspond to holes and electrons, respectively.

The impedance spectra were analyzed using the equivalent circuit approach. We have assumed that there are the following two areas formed within the SY layer when the electric field is applied: one with electrons prevailing, and the other with the holes as the main charge carrier. The recombination between those two types of charge carriers takes place within a thin layer between both of the areas. The hole-enriched area starts at the anode on the interface PEDOT:PSS/SY and extends towards the recombination interface, while the layer enriched with electrons is located on another side of the recombination interface, formed by electrons coming from the calcium cathode direction. The two areas are attributed to the following two elements of the equivalent electrical circuit: *Z*_1_ (zone 1) and *Z*_2_ (zone 2). One can suppose that the processes responsible for the registered AC response occur within the SY layer. The total resistance of the electrical contacts and PEDOT:PSS layer is represented in the model as an Rs element. The equivalent circuit proposed before [13] (Figure 3c) fitted all the experimental spectra. The impedance frequency (*Z-ω*) function of the model is given by the following:

(2) $$\begin{array}{c} Z=R_{s}+Z_{1}+Z_{2}\\ \mathrm{for}:\;\;Z_{1}=\frac{R_{1}}{1+j\omega \tau_{1}}\;\mathrm{and}\;Z_{2}=\frac{R_{2}}{1+j\omega \tau_{2}} \end{array}$$

where *τ*_1_ and *τ*_2_ are the time constants of the processes, which are responsible for the dynamics of the rearrangement of the electric charges. The five equivalent circuit parameters *R*_1_, *R*_2_, *Rs*, *τ*_1_ and *τ*_2_ were calculated for every DC voltage applied. Their voltage dependencies are shown in Figure 4a,b.

One may notice that at *U_DC_* < 1.9 V, the resistance *R*_2_ (resistance of zone 2) is more than ten times larger than the resistance *R*_1_. Such an asymmetry can be related to the differences between the mobility of the charge carriers, or be reasoned by an energetic barrier (Δ*E*) for the injection of one type of charge carrier at one of the interfaces electrode/organic layer.

**Figure 5:**
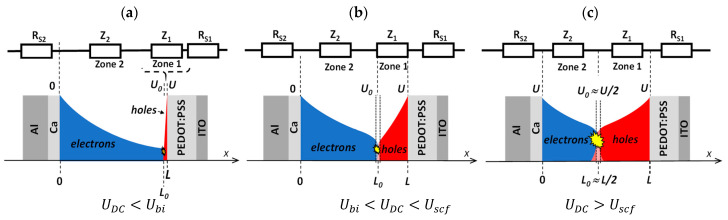
Distribution of the density of holes (Zone 1) and electrons (Zone 2) in the Super Yellow layer with thickness *L* for the case where bias voltage *U* is: lower than built-in voltage (**a**), higher tan built-in voltage and lower than space charge formation voltage (**b**), higher than space charge formation voltage (**c**). At the top are the equivalent circuit parameters of PLED corresponding to Zone 1, Zone 2, PEDOT:PSS/ITO electrode and Al/Ca electrode. The symbols *L*_0_ and *U*_0_ denote the location and voltage of the recombination front, respectively. The bright yellow shape indicate a recombination front where electroluminescence is mainly taking place.

It is known from the literature that the mobilities of the holes and electrons are almost equal in the SY [9], and there is a barrier Δ*E* equal to about 0.3 V at the PEDOT:PSS/SY interface (Figure 1). Thus, the density of the injected holes should be noticeably smaller than that of electrons, and the recombination interface should be localized in the vicinity (at the coordinate *L*_0_, Figure 5) to the PEDOT:PSS/SY interface. In that case, zone 2 filled with electrons would extend to almost a whole SY layer, while zone 1 filled with holes would appear in a narrow layer at the SY/Ca interface (Figure 5a).

The voltage between the cathode and the recombination interface can be estimated based on the relation *U*_0_ = *KU_DC_*, where the following applies:

(3) $$K=\frac{R_{2}}{R_{1}+R_{2}}$$

Figure 4c shows that at *U_DC_* < 1.9 V, the coefficient of the voltage division *K* remains close to unity. In that case, a relatively small drift and diffusion current (DDC) flows through the organic layers. The current almost linearly depends on the applied voltage (Figure 2). As the voltage grows, the drift current decreases, while the diffusion current increases [14].

Due to the appearing diffusion current, the resistances *R*_1_ and *R*_2_ decrease at the first stage. Especially when *U* approaches *U_bi_,* the drop is most noticeable. As the width of zone 2 is significantly larger than the volume of zone 1, the time required to deliver the electrons from the electrode to the recombination region is longer than the time needed for the holes to reach the recombination interface, *τ*_2_ > *τ*_1_, as Figure 5a illustrates. At *U_DC_ = U_bi_ ≈* 1.9 V, the diffusion current reaches its maximum value, while the drift current falls down to zero.

A further increase in the voltage is accompanied by drastic current and luminance growth (Figure 2a). In that case, the drift current changes its direction and becomes considerably higher, starting to dominate over the diffusion current [15]. Figure 4 clearly shows a decrease in the voltage divide coefficient *K* along with the resistances *R*_1_ and *R*_2_, and time constants *τ*_1_ and *τ*_2_. The reason for the observed drastic changes lie upon the following phenomena: the formation of the recombination interface, its shift towards the middle of the SY layer, enhancement of the mobility due to the density of states filling [16,17], and increasing efficiency of the emissive recombination between the holes and electrons (Figure 5b). The resistances *R*_1_ and *R*_2_ decrease due to the increase in the charge carriers density. The time constants *τ*_1_ and *τ*_2_ drop down as well due to the acceleration of the charge carriers in zones 1 and 2, favored by facilitated recombination. The resistances and time constants of both of the zones achieve similar values, mainly because of the equalizing of both of the charge path lengths (widths of the zones) (Figure 5c).

At a voltage higher than 2.7 V, denoted below as a space charge formation voltage, *U_scf_*, the system becomes symmetrical (Figure 5c). The *K* coefficient value close to $\frac{1}{2}$ and at almost equal time constants (Figure 4) confirms that conclusion.

It is worth mentioning that a further analysis for higher voltages can give only joint resistance *R* = *R*_1_ + *R*_2_ and a joint time constant value *τ* = *τ*_1_ = *τ*_2_. In the case when the time constants are equal *τ* = *τ*_1_ = *τ*_2_ there is no theoretical possibility to calculate all four values, as the Formula (2) could be simplified to (4), where values *R*_1_, *R*_2_, *τ*_1_ and *τ*_2_ are inseparable.

(4) $$Z=R_{s}+\frac{R}{1+j\omega \tau }$$

That case makes an unsolvable problem in impedance analysis, when *τ*_1_ and *τ*_2_ are close.

The space charge regions formed in zones 1 and 2 at voltages *U* > *U_scf_* affect the intrinsic electric field in the semiconductor. In that case, equal hole (*j_hSCLC_*) and electron (*j_eSCLC_*) currents limited by the space charge flow through the semiconductor. The magnitude of both of the currents is described by the Mott–Gurney equation [18].

(5) $$\;j_{SCLC}=j\left(U_{scf}\right)+\frac{9}{8}\frac{\mu \varepsilon \varepsilon_{0}}{{\left(L/2\right)}^{3}}{\left(\frac{U-U_{scf}}{2}\right)}^{2}$$

where *ε* is the dielectric constant, *ε*_0_ is the electric constant, *L* is the semiconductor thickness and *μ* is the mobility of the charge carriers (electrons or holes).

The mobility of the holes and electrons, calculated by fitting the experimental data presented in Figure 2 by Equation (5), equals *μ_e_* ≈ *μ_h_* ≈ 1.1·10^−5^ cm^2^V^−1^s^−1^ (the dielectric constant equal to 3 was accepted for the calculations). The values are in accordance with the results obtained by other authors using the transient electroluminescence method [19].

An experienced PLED researcher is aware of the difficulties accompanying the fabrication of a highly stable PLED. Despite the quite clear and not sophisticated procedure, the minor, least noticeable deviation from the protocol would cause device degradation within the first several weeks of operation. The following subsection is dedicated to showing what happens with an unprotected device by taking an insight into charge transport on a macromolecular scale.

### 3.2. The PLEDs Device without Protecting Layer Fast Degradation

**Figure 6:**
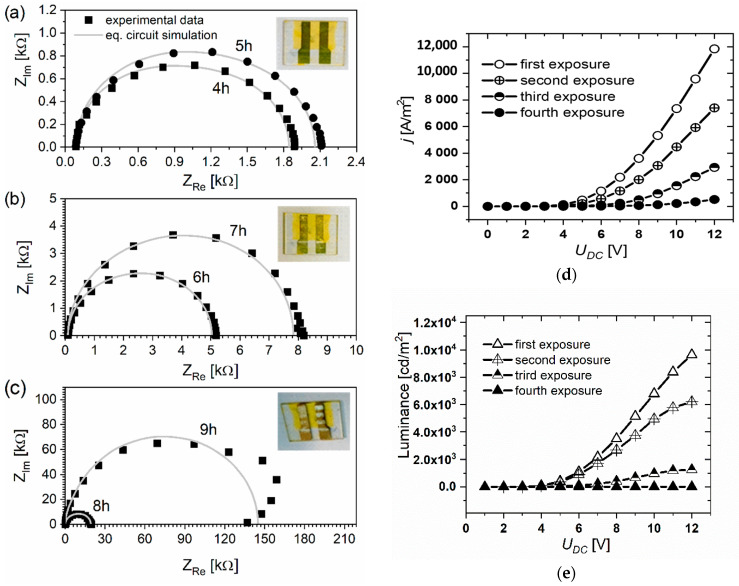
Degradation of PLED caused by the ambient atmosphere. Nyquist plots at operating voltage 9V (**a**–**c**), current-voltage characteristic (**d**), luminance as a function of voltage (**e**).

As was expected, the lack of protecting foil made the device vulnerable to the natural environment. Figure 6 demonstrates the evolution of the impedance spectra of a PLED exposure to the ambient atmosphere.

As soon as being taken outside the glovebox, a PLED starts degrading drastically. The process is evidenced by the fast growth of the impedance, decrease in DC conductivity (Figure 6d), and loss of luminescent properties (Figure 6e). Impedance spectroscopy allows to obtain information about the changes in the interfaces caused by the degradation of the device during electrical operation [20]. IS allows to effectively monitor the changes in the electrical parameters of organic devices over time, caused by the action of water or oxygen molecules [21].

The increase in impedance is so great that the data cannot be plotted on one graph (Figure 6a–c, mind the scale). The destruction of the device is perceivable with an unaided eye (insets in Figure 6a–c).

**Figure 7:**
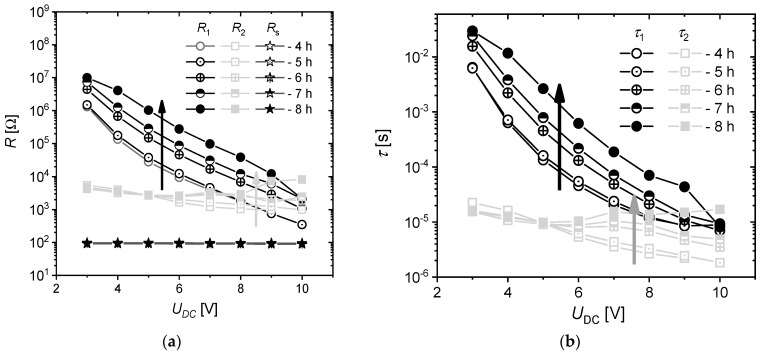
Evolution of equivalent electrical circuit parameters with time and DC voltage; resistors (**a**); time constants (**b**). Vertical arrows indicate the observed tendency of parameter change with time.

The resistances and time constants were obtained from impedance spectra as a function of applied voltage and elapsed time (Figure 7).

Due to the significant differences in the parameters measured at the different elapsed times, the data are represented on a logarithmic scale. The time constants of both types of charge carriers have been calculated in the same way as discussed in Section 3.1.

An analogous study of the unprotected diode included an additional parameter—the time elapsed since the diode was exposed to the ambient atmosphere. The tendencies of the parameter changes with voltage (Figure 7a,b) remain similar to each other and those of the protected device (Figure 4a,b).

At voltages higher than 5 V, the time constants related to the electrons increased with time, i.e., the ETL quickly loses conductivity without encapsulation.

### 3.3. The PLEDs with Damaged Encapsulating Cover Slow Degradation

**Figure 8:**
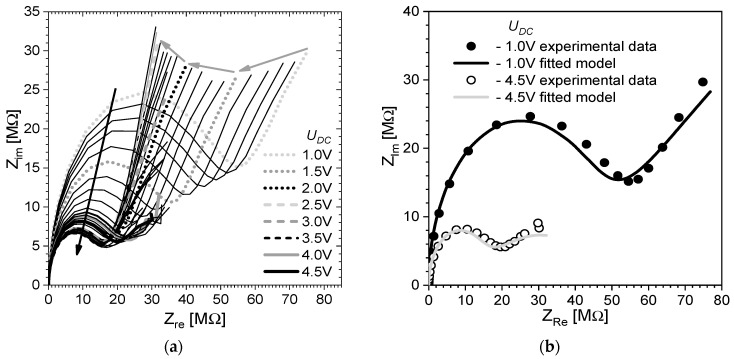
Set of impedance spectra of a malfunctioned PLED after twenty days in the applied DC voltage range from 1.0 V to 4.5 V (**a**) and example of fitting the spectra at 1.0 V and 4.5 V with equivalent circuit model (**b**). The arrows indicate the change in the spectra with voltage.

The following two limited cases have been considered above: the ideally protected device and the device without protection. Nevertheless, in most cases a researcher encounters the situation in between those cases—the device works normally during the first several hours, enabling to carry out all the measurements, but loses its properties within several days. To study the harmful process responsible for the slow degradation of an encapsulated device, we have deliberately prepared a diode with a partially destroyed protective cover. The encapsulation foil of a specimen diode was deliberately notched with a needle with the intention to observe the evaluation of the conductive characteristics affected by chemically active compounds from the environment. A gradual decrease in device conductivity was observed until the complete loss of the electroluminescent properties. Despite that, the device retained conductivity, with a considerable change in the impedance spectrum observed. The impedance spectra of the device with the faulted protecting cover, after twenty days of exposure to the ambient atmosphere, have a form of a quasi-semicircle with a low-frequency straight tail (Figure 8a). One has to pay attention to the very high values of impedance, which indicate that the charge transport through the organic layers is obstructed. Even if the active molecules retain their luminescent properties, the amount of delivered charge is too limited to enable device operation. The semicircle form of the impedance spectra is a common effect. In the analysis, it is usually regarded as a shunted capacitor. The straight low-frequency part sloped 45° to the axis (given that scales are equal), which is regarded as a diffusion process indicator in analogy to the electrochemical processes limited by diffusion. However, in the case of organic devices, the phenomenon is much more complicated. Two of the analyzed spectra in Figure 8b are presented in an equal-scaled plot. One can see that the semicircle is distorted and the low-frequency tail has a different slope at different voltages, but not equal to 45° to the axes.

**Figure 9:**
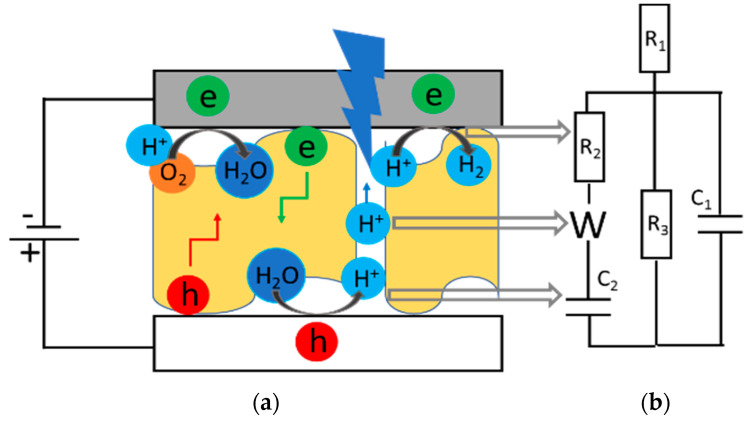
Schematic illustration of charge-transport processes in operational PLED and device after partial destruction (**a**). Figure 3. O^+^) are designated as H^+^. Equivalent electrical circuit (**b**).

**Figure 10:**
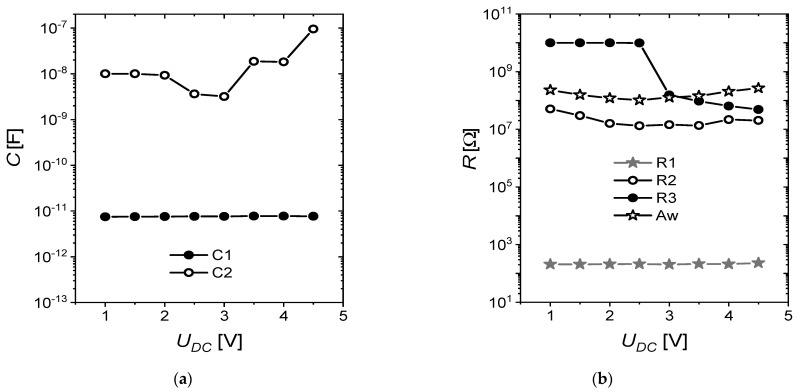
Dependence of equivalent circuit parameter values on the applied DC voltage. Capacitance (**a**), and resistance and Warburg coefficient A_W_ (measure unit Ω × s^−1/2^) (**b**).

We have worked out another model that could be used for the analysis of the spectra and interpretation of the observed phenomenon. The proposed equivalent circuit and corresponding processes responsible for charge transport in the destructed device are shown in Figure 9. The values of the calculated equivalent circuit parameters are shown in Figure 10.

The physical meaning of the parameters and the destructing chemical processes will be discussed below.

The partial destruction of the protective layer leads to disturbance of the contact of the organic layers with ITO and a metal. Therefore, the conductivity of the layer drops down, and the only charge exchange between the electrodes is possible due to electroactive components, which are presented in the internal layer (H^+^ in PEDOT:PSS) or come from the environment (H_2_O and O_2_).

The charge transfer at the anode (ITO) surface may occur due to water oxidation (R1).

2H_2_O − 4e = 4H^+^ + O_2_ (R1)

The cation H^+^ is unlikely to be able to flow inside the solid medium. In the case of the presence of traces of water, a hydronium cation (H_3_O^+^ = H_2_O∙H^+^) is formed, which could diffuse into an aqueous medium towards the cathode.

The second option for the delivery of positive charge from the anode is ensured by PEDOT. The scheme shown below illustrates the transport of a positive charge from the electrode (El) to the end of the conjugated polymer chain.


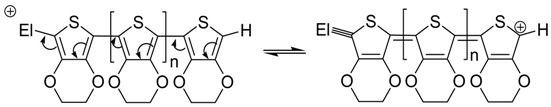


When the device is working correctly, the delivered charge recombines with the electron at the interface between hole-transport and electron-transport layers. However, in the case of excess water inside the film, the charge could be intercepted according to scheme (R2).

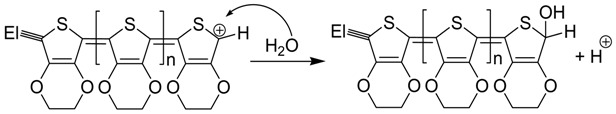
(R2)


The charge exchange at the cathode (Al/Ca- layer) may occur due to the following processes:

2H^+^ + 2e = H_2_ (R3)

2H_2_O + 2e = H_2_ + 2OH^−^ (R4)

O_2_ + 4H^+^ + 4e = 2H_2_O (R5)

O_2_ + 2H_2_O + 4e = 4OH^−^ (R6)

The relative impact of each process on the total current depends on the presence of the corresponding reagents. Oxygen as a strong oxidant is expected to play an important role in accepting the charge from the cathode (half-reactions R5 and R6). In the case when the delivery of hydronium ions from the anode is effective, reaction R5 is preferable. Otherwise, hydroxide ions are formed predominantly (R6). They further diffuse to the anode, and somewhere on the path they meet and neutralize the hydrogen ions to form water.

The described hypothesis about the processes occurring inside the diode is confirmed by the voltage dependences of the equivalent circuit parameters. The first capacitance C_1_ (Figure 10a) representing the detached branch in the equivalent circuit (Figure 9b) represents weak capacitive properties of the diode, which remained despite diode destruction and shunting. The value slightly increases with the voltage due to the polarization and accumulation of charges.

The three-element branch recalls the famous Frumkin–Melik–Gaikazian equivalent circuit [13], proposed for electrochemical reactions involving both diffusion and adsorption. The capacitance C_2_ (Figure 10a) could be attributed to the accumulation of the charge species near the electrodes, either the cathode or anode. The anode is more favorable as it is located closer to the breach of the protective layer. Resistance R_2_ (Figure 10b) demonstrates a noticeable fall caused by polarization, suggesting that it could be related to the electrochemical process. The same effect is observed for Warburg coefficient A_W_ (Figure 10b), which is related to diffusion. The diffusion rate is the highest (when the Warburg impedance is the smallest) at about 2.5 V. The results on R_2_ and A_W_ indicate obstruction of the charge path at very high voltages. Resistance R_3_ could not even be detected within the low voltage range (Figure 10b)—the value of 10 GΩ is the detection limit, but becomes apparent and is necessary for fitting the model at high voltages. We attribute this element to the transport of charged species driven by the electric field. Finally, resistance R_1_ (Figure 10b) corresponds to all non-electrochemical resistances of the measurement setup.

## 4. Conclusions

The stability of a model PLED based on Super Yellow—the most often used polymer as an emission layer in PLEDs [22]—has been studied in terms of the conductive–capacitive properties of the organic layers. It was found that the AC response is extremely sensitive to the minor changes in PLED caused by the applied electric field, and contact with the ambient atmosphere. In particular, microscopic damages or breach’s in the electric current paths are detected by an increase in resistance and a decrease in capacitance, i.e., the loss of both conductive and capacitive properties of the organic layer. The changes observed for an unprotected device were about two orders of magnitude within the first 8 h of device exploitation. Due to different charge mobilities, the characteristics of the internal layers were studied individually to monitor the ageing of the particular device components. Within the presented work, three cases of destruction have been considered from the experimental and theoretical point of view. A universal strategy for the analysis and interpretation of the impedance data has been presented.

## Data Availability

The data presented in this study are available on request from the corresponding author.
